# Plasma miRNAome Profiling Reveals Candidate Biomarkers for Low- and High-Dose Whole-Body Ionizing Radiation Exposure

**DOI:** 10.1177/15593258251391602

**Published:** 2025-10-23

**Authors:** Gizelle J. Lionel, Ronan Derbowka, Shayen Sreetharan, Jocelyn Bel, Jessica Dougherty, Douglas R. Boreham, T.C. Tai, Christopher Thome, Simon J. Lees, Sujeenthar Tharmalingam

**Affiliations:** 1 School of Natural Sciences, 7728 Laurentian University, Sudbury, ON, Canada; 2 Medical Sciences Division, 26627 NOSM University, Sudbury, ON, Canada; 3 Department of Physics and Astronomy, McMaster University, Hamilton, ON, Canada; 4 Department of Biology, 7890 Lakehead University, Thunder Bay, ON, Canada; 5 Health Sciences North Research Institute, Sudbury, ON, Canada

**Keywords:** radiation exposure, miRNA biomarker, miRNAome, biodosimetry, next-generation sequencing, low-dose radiation, high-dose radiation, plasma miRNA profiling, mouse model

## Abstract

**Objective:**

MicroRNAs (miRNAs) are small, non-coding RNA molecules that regulate gene expression and remain stable in biological fluids, even under harsh conditions. Their stability and responsiveness to environmental stressors make them strong candidates for radiation biodosimetry. This study aimed to (1) establish a robust in vivo pipeline for miRNAome profiling and (2) identify plasma-based miRNA biomarkers of ionizing radiation at low and high doses.

**Methods:**

BALB/c mice were exposed to sham, 100 mGy, or 2 Gy of X-rays. Plasma was collected 6 h post-irradiation. Total RNA was extracted, and next-generation sequencing was used to profile the plasma miRNAome. Differentially expressed miRNAs were identified relative to sham controls, and selected candidates were validated using RT-qPCR.

**Results:**

A total of 630 unique miRNAs were detected. High-dose exposure (2 Gy) significantly upregulated 14 and downregulated 5 miRNAs. Seven miRNAs were significantly induced at 100 mGy, including miR-126a-5p and miR-133a-3p, which were exclusive to low-dose exposure. Five miRNAs were shared between both doses, indicating dose-independent responses. RT-qPCR confirmed expression trends.

**Conclusion:**

This study identified distinct and shared circulating miRNA signatures for low- and high-dose radiation exposure. These findings support the potential of miRNAs as minimally invasive, dose-stratified biomarkers for radiation biodosimetry.

## Introduction

Biodosimetry is the quantitative assessment of biological responses to ionizing radiation and plays a critical role in estimating exposure levels following accidental or occupational radiation incidents.^1^ The current gold standard in radiation biodosimetry is the dicentric chromosome assay (DCA), which detects dicentric chromosomes in peripheral blood lymphocytes.^2^ While the DCA is highly reliable for high-dose radiation (HDR) exposures, it exhibits considerable limitations at low-dose radiation (LDR) ranges (below 100 mGy), with a typical predictive error of ±0.5 Gy.^3^ The cytokinesis-block micronucleus (CBMN) assay, another widely used biodosimetry technique that measures micronuclei formation, also suffers from reduced sensitivity at low radiation doses and limited effectiveness in detecting partial-body exposures.^4^ Both techniques are low-throughput, require specialized personnel, and depend on mitotically active cells, limiting their application in large-scale or rapid screening scenarios.^5,6^

There is a growing need for biodosimetry tools that are accurate, sensitive to LDR, high-throughput, and minimally invasive. MicroRNAs (miRNAs) have emerged as promising biomolecules for radiation biodosimetry due to their abundance, accessibility in blood-based biofluids, and remarkable biochemical stability.^7,8^ These small, non-coding RNAs (∼20-24 nucleotides in length) regulate gene expression post-transcriptionally by guiding the RNA-induced silencing complex (RISC) to degrade or repress the translation of target mRNAs.^9,10^ Mature miRNAs are derived from primary transcripts (pri-miRNAs) through a multistep biogenesis pathway, with one of the two mature products—either the 3p or 5p strand—preferentially incorporated into the RISC complex.^11^

miRNAs influence critical cellular responses to radiation, such as the DNA damage response (DDR), apoptosis, and cell-cycle regulation.^12-14^ For instance, radiation-induced DNA double-strand breaks (DSBs) typically activate the ATM kinase pathway, leading to phosphorylation of key DDR proteins and recruitment of repair factors such as BRCA1.^15,16^ Notably, miR-125b-5p directly regulates BRCA1 by binding its 3' untranslated region, suppressing BRCA1 protein levels.^17^ Elevated miR-125b-5p following radiation thus impairs DNA repair, increasing genomic instability and sensitizing cells to apoptosis. This illustrates how miRNAs directly influence radiation response pathways, highlighting their value as precise biomarkers for biodosimetry.

miRNAs are stably present in plasma and other body fluids and remain intact even after exposure to extreme pH changes, enzymatic degradation, and repeated freeze–thaw cycles.^18-20^ While numerous studies have demonstrated significant alterations in miRNA expression following HDR in cancer models or irradiated cell lines,^21-23^ relatively few have explored the miRNAome response to LDR using unbiased next-generation sequencing (NGS) approaches in vivo. Notable examples include recent mouse studies reporting plasma or exosomal miRNA signatures following whole-body or tissue-specific LDR exposures,^24,25^ though such efforts remain sparse compared to HDR investigations. Moreover, most prior studies have relied on candidate miRNA screening methods, which can limit discovery of novel dose-dependent miRNA changes.

To address these gaps, we employed an NGS-based miRNAomics workflow to comprehensively profile the plasma miRNAome of *BALB/c* mice following whole-body exposure to either 100 mGy or 2 Gy of X-rays. Plasma samples were collected 6 h post-irradiation and analyzed to identify dose-dependent changes in circulating miRNAs. This study builds on our previously established NGS-based workflow for miRNAome profiling in cultured cells exposed to ionizing radiation,^26^ extending the application of this platform to in vivo models. The primary aims of this study were to (1) establish a robust pipeline for in vivo miRNAome profiling and (2) identify novel plasma-based miRNA biomarkers of ionizing radiation exposure at both low and high doses. Our findings support the utility of miRNA-based biodosimetry and identify candidate plasma miRNAs with potential diagnostic value for future radiation exposure screening.

## Materials and Methods

### Animals

Wild-type male BALB/cAnNCrl mice were obtained from Charles River Laboratories (Saint-Constant, Quebec, Canada). All mice were 4 months of age at the time of irradiation. Animals were singly housed under standard conditions and provided ad libitum access to food and water for the duration of the study, except during irradiation procedures. All protocols were reviewed and approved by the Lakehead University Animal Care Committee (Animal Use Protocol #1467646).

BALB/c mice were selected because they are one of the most commonly used inbred strains in radiation biology and immunology, with well-characterized radiosensitivity (LD_50/30_ ∼5000 mGy).^27-29^ This value is closer to the human LD_50/30_ (∼4500 mGy^30^) than that of more radioresistant strains such as C57Bl/6 (∼6300 mGy), making BALB/c a translationally relevant model for both low- and high-dose radiation studies.^31^ Their radiosensitive background also facilitates the detection of subtle biological responses, which is advantageous for biomarker discovery.

### Irradiations

Irradiations were conducted in the animals’ home cages to minimize stress during exposure. Food and enrichment materials (eg, animal igloo and nesting material) were removed prior to irradiation to reduce variability in absorbed dose. Mice were transported from the housing room to the irradiation room—located within the same facility—using a lab cart. Whole-body X-ray exposures were performed using an X-Rad320 cabinet irradiator (Precision X-Ray Inc, Madison, CT, USA) at doses of 100 mGy, 2 Gy, or sham irradiation (5 animals per condition). Irradiation parameters were as follows: 320 kVp, 12.5 mA, and a dose rate of 0.364 Gy/min. All treatment groups remained inside the irradiator for an equal duration (5 minutes and 25 seconds), corresponding to the time required to deliver the 2 Gy dose. Sham-irradiated animals were transported and placed in the irradiator under identical conditions, with the X-ray beam powered off. Absorbed doses were verified using thermoluminescent dosimeters (Mirion Technologies, Atlanta GA, USA).

### Tissue Collection

A 6-h post-irradiation interval was selected based on prior in vitro studies,^26^ which demonstrated maximal radiation-induced miRNA responses at this timepoint compared to 1 h or 24 h. At this 6-hour interval, mice were anesthetized under terminal isoflurane anesthesia. Needles and syringes were pre-coated with cold 0.5 M EDTA (pH 8.0) to prevent coagulation. Approximately 400 µL of blood was collected via cardiac puncture using a 22G, 1-inch needle. Blood was immediately dispensed into 1.5 mL microcentrifuge tubes, gently inverted five times, and stored on ice prior to transport to the wet laboratory facility. All samples were processed within 2 h of collection. Blood was centrifuged at 2000 × g for 10 min at 4 °C. Plasma was carefully transferred to new 1.5 mL tubes and stored at −80 °C until further analysis.

### miRNA Extraction

Total RNA, including small RNA species, was extracted from 100 µL of plasma using the miRNeasy Serum/Plasma Kit (QIAGEN, Germantown, MD, USA; Cat. No. 217184), following the manufacturer’s protocol. Briefly, the protocol uses QIAzol Lysis Reagent for efficient denaturation of protein–RNA complexes, followed by chloroform-based phase separation and silica-membrane column purification of total RNA. RNA was eluted in 14 µL of RNase-free water, yielding a preparation enriched in total and small miRNAs. All samples were stored at −80°C until further processing.

### miRNAome Profiling via Next-Generation Sequencing (NGS)

miRNAome profiling was conducted using 5 µL of purified RNA obtained from 100 µL of plasma. Library preparation was performed using the QIAseq miRNA Library Kit (QIAGEN, Cat. No. 331502), which selectively enriches small RNA species and prepares them for sequencing. Briefly, adapter sequences were ligated to the 5′ and 3′ ends of miRNAs, followed by reverse transcription to generate cDNA incorporating unique molecular identifiers (UMIs). A final PCR amplification step was used to introduce sample-specific barcodes. Once libraries were prepared, library concentration was verified using the NEBNext® Library Quant Kit for Illumina® (New England Biolabs, Cat. No. E7630) according to the manufacturer’s instructions. Libraries were then pooled at equimolar concentrations, and the pooled sample was assessed using a Bioanalyzer (Agilent Technologies) at the McMaster Genomics Facility (Hamilton, ON, Canada) to confirm an expected library size of approximately 180 bp. Sequencing was performed on the Illumina NextSeq 2000 platform using a 1 × 100 bp configuration, targeting a depth of 10 million reads per sample. FASTQ files obtained from the sequencer were uploaded to the QIAGEN Data Analysis Center (https://www.qiagen.com/data), where primary data analysis was conducted. UMIs were counted, and miRNA sequences were mapped to the reference database. Secondary analysis was then performed using UMI-based miRNA counts, and differential expression analysis was completed by the software to generate final expression results.

### RT-qPCR

Reverse transcription quantitative polymerase chain reaction (RT-qPCR) was used to validate selected miRNAs using prevalidated miRCURY LNA miRNA PCR Assays (QIAGEN). The following miRNAs were assessed, with their corresponding prevalidated primer mix catalog numbers: *miR-126a-5p* (QIAGEN, YP00206010), *miR-133a-3p* (QIAGEN, YP00204788), *miR-2137* (QIAGEN, YP00205680), *miR-99a-5p* (QIAGEN, YP00204521), and *miR-126a-3p* (QIAGEN, YP00205682). Two endogenous control miRNAs were included based on stable expression across samples: *miR-423-5p* (QIAGEN, YP00205624) and *miR-16-5p* (QIAGEN, YP00205624).

cDNA synthesis was performed using 5 µL of total RNA extracted from 100 µL of plasma, using the *miRCURY LNA RT Kit* (QIAGEN, Cat. No. 339340). A master mix was prepared by combining 9 µL of RNase-free water, 4 µL of 5X *miRCURY SYBR Green RT Reaction Buffer*, and 2 µL of 10X *miRCURY RT Enzyme Mix*. A total of 15 µL of master mix was added to 5 µL of RNA per reaction. Reverse transcription was carried out at 42°C for 60 minutes, followed by heat inactivation at 95°C for 5 minutes and cooling to 4°C. The resulting cDNA was diluted 1:30 by adding 58 µL of RNase-free water to 2 µL of the RT product.

qPCR reactions were prepared using MediRes Bi2M qPCR 2X Master Mix (Cat. No. Bi2M-2xqPCR). A master mix was assembled using 7.5 µL of 2X *Master Mix*, 1.5 µL of resuspended miRNA PCR primer mix, and 1 µL of RNase-free water. For each reaction, 10 µL of master mix was combined with 5 µL of diluted cDNA. Reactions were mixed, centrifuged briefly, and run on the QuantStudio 5 Real-Time PCR instrument (Thermo Fisher Scientific) using the following cycling conditions: 95°C for 2 minutes, followed by 40 cycles of 95°C for 10 seconds and 56°C for 60 seconds.

The relative expression of each miRNA was calculated using the ΔΔCT method,^32^ based on the formula: 2^ΔΔCT^ = 2^(ΔCT gene −^ ^ΔCT housekeeping genes)^. The average 2^ΔΔCT^ and standard error of the mean (SEM) were calculated for each miRNA.

### Statistical Analysis

For the NGS data, irradiated samples were first normalized to their respective sham controls using the QIAGEN Data Analysis Center, as previously described.^26^ miRNAs were considered significantly differentially expressed if they met all of the following criteria: *P* < 0.05, false discovery rate (FDR) < 0.1, expression level ≥50 transcripts per million (TPM), and fold change ≥1.5. In addition to sham-referenced comparisons, we tested the 2 Gy vs 100 mGy contrast using the same thresholds.

For RT-qPCR, group differences were assessed by one-way ANOVA with Tukey’s post-hoc test across sham, 100 mGy, and 2 Gy. Significance was reported vs sham (*, *P* < 0.05) and for the 100 mGy vs 2 Gy pairwise comparison (#, *P* < 0.05). Statistical analyses were conducted using Jamovi (version 2.3.28). A *P*-value <0.05 was considered statistically significant.

## Results

### Profiling of Circulating miRNAs Following High-Dose Radiation Exposure

**Figure 1.**
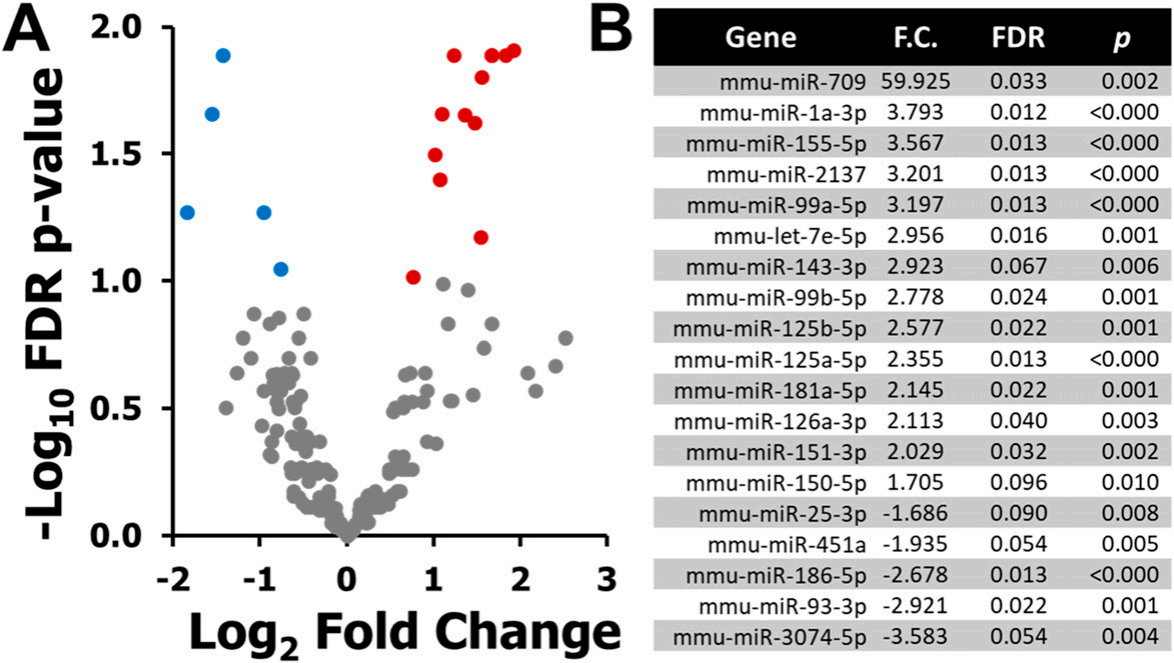
Differentially Expressed Plasma miRNAs 6 h after 2 Gy X-Ray Exposure in BALB/C Mice. (A) Volcano Plot Showing log_2_ Fold Change Versus –log_10_ FDR *P*-value for all 630 Detected Plasma miRNAs. Differential Expression was Defined by the Following Criteria: *P* < 0.05, False Discovery Rate (FDR) < 0.1, Expression Level ≥50 Transcripts per Million (TPM), and Absolute Fold Change ≥1.5. Red Dots Indicate Significantly Upregulated miRNAs, Blue Dots Indicate Significantly Downregulated miRNAs, and Grey Dots Represent Non-significant Changes. (B) List of the 19 Differentially Expressed miRNAs Identified in the 2 Gy Versus Sham Comparison, including Gene Name, Fold Change (F.C.), FDR, and Ranked Based on F.C

To investigate miRNA responses to ionizing radiation, the plasma miRNAome of BALB/c mice exposed to 2 Gy X-ray was profiled 6 h post-irradiation using NGS. A total of 630 unique plasma miRNAs were detected across samples. Differential expression analysis identified 19 miRNAs that met the filtering criteria (FDR <0.1, *P* < 0.05, ≥50 TPM, fold change ≥1.5). Of these, 14 miRNAs were significantly upregulated, while 5 were significantly downregulated compared to sham-treated controls (Figure 1A). The top upregulated miRNAs included miR-709, miR-1a-3p, miR-155-5p, miR-2137, and miR-99a-5p, whereas miR-25-3p, miR-451a, miR-186-5p, miR-93-3p, and miR-3074-5p were among the downregulated transcripts (Figure 1B).

### Low-Dose Radiation Elicits a Distinct Circulating miRNA Signature

**Figure 2.**
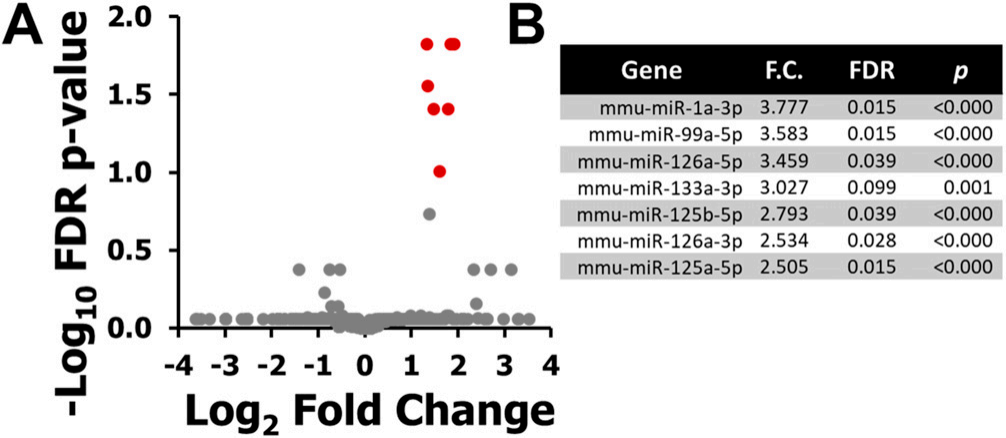
Differentially Expressed Plasma miRNAs 6 h after 100 mGy X-Ray Exposure in BALB/C Mice. (A) Volcano Plot Showing log_2_ Fold Change Versus –log_10_ FDR *P*-value for all Detected Plasma miRNAs. Differential Expression was Defined by the Following Criteria: *P* < 0.05, False Discovery Rate (FDR) < 0.1, Expression Level ≥50 Transcripts per Million (TPM), and Absolute Fold Change ≥1.5. Red Dots Indicate Significantly Upregulated miRNAs, and Grey Dots Represent Non-significant Changes. (B) List of the 7 Differentially Expressed miRNAs Identified in the 100 mGy Versus Sham Comparison, including Gene Name, Fold Change (F.C.), FDR, and Ranked Based on F.C

Low-dose radiation exposure (100 mGy X-ray) also triggered a specific miRNA expression response in mouse plasma (Figure 2A). A total of 7 miRNAs were significantly upregulated relative to sham (Figure 2B), with no downregulated miRNAs identified under these criteria. MiRNAs induced at this dose included miR-1a-3p, miR-99a-5p, miR-126a-5p, miR-133a-3p, miR-125b-5p, miR-126a-3p, and miR-125a-5p.

### Shared and Dose-specific miRNA Expression Patterns

**Figure 3.**
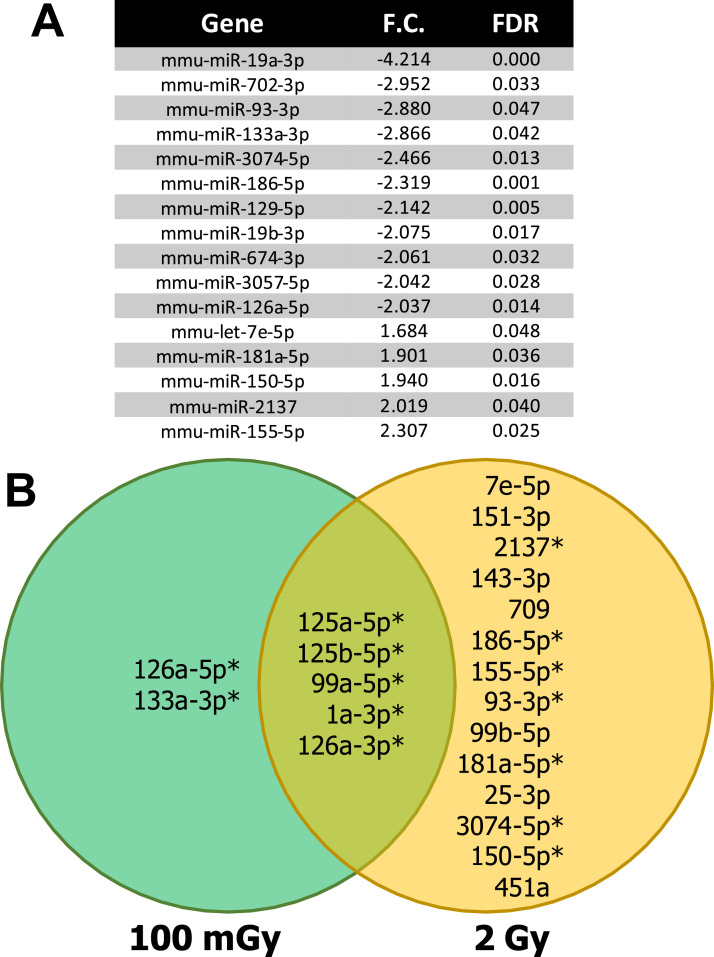
Cross-Dose Differential Expression and Overlap of Plasma miRNAs 6 h after Whole-Body X-Ray Exposure in BALB/C Mice. (A) Differential Expression in the 2 Gy vs 100 mGy Comparison, Ranked by Fold Change (Most Decreased to Most Increased in 2 Gy Relative to 100 mGy). Significance Thresholds: False Discovery Rate (FDR) < 0.10, Expression ≥50 Transcripts per Million, and Fold Change ≥ or ≤1.5. (B) Venn Diagram Showing the Overlap of Differentially Expressed miRNAs Identified in 100 mGy vs Sham (Figure 2) and 2 Gy vs Sham (Figure 1). MiRNA Names are Shortened by Removing the “Mmu-miR” Prefix. An Asterisk (*) Denotes miRNAs Whose Direction and Significance are Concordant Across Both Sham-Referenced Results (Figures 1 and 2) and the 2 Gy vs 100 mGy Comparison in Panel A

To address whether sham-referenced differences translate into separation between LDR and HDR groups at 6 h, we compared the miRNAome datasets between 2 Gy vs 100 mGy using the same stringency thresholds: false discovery rate ≤0.10, minimum abundance ≥50 transcripts per million, and fold change ≥ or ≤1.5 (Figure 3A). This analysis identified significant between-dose differences for a subset of miRNAs, presented ranked by fold change (2 Gy relative to 100 mGy). Since LDR-vs-HDR results are most meaningful when the candidate miRNAs are also perturbed relative to baseline, we interpreted these findings in concert with the sham-anchored results below.

A Venn diagram (Figure 3B) was constructed to compare the overlap of differentially expressed miRNAs between the 100 mGy vs sham (Figure 2) and 2 Gy vs sham (Figure 1) results. To better capture overlapping dose-dependent signals, miRNAs with concordant direction and significance in the 2 Gy vs 100 mGy comparison are marked with an asterisk in the Venn. Using this double-referencing, we find five miRNAs significantly dysregulated in both LDR and HDR conditions: miR-1a-3p, miR-99a-5p, miR-125a-5p, miR-126a-3p, and miR-125b-5p. We also confirm that the LDR-specific miRNAs identified relative to sham, miR-126a-5p and miR-133a-3p, are significantly different between LDR and HDR. Finally, among the 14 miRNAs that were differentially expressed in the 2 Gy vs sham group, 7 show concordant differences between 2 Gy and 100 mGy: miR-7e-5p, miR-151-3p, miR-2137, miR-143-3p, miR-709, miR-186-5p, and miR-155-5p. Taken together, miRNAs that are significant vs sham and concordant between doses represent the strongest candidate features for dose binning and should be prioritized for validation in larger, independent cohorts.

### RT-qPCR Validation of Candidate miRNAs

**Figure 4.**
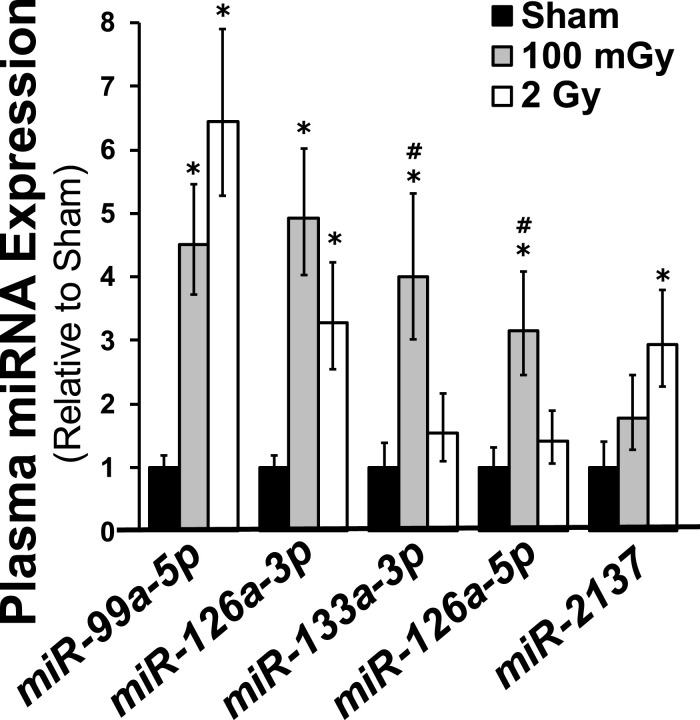
RT-qPCR Validation of Select Differentially Expressed Plasma miRNAs Following Ionizing Radiation Exposure in BALB/C mice. Expression Levels of Five miRNAs Were Assessed by RT-qPCR 6 h after Sham, 100 mGy, or 2 Gy X-Ray Exposure. The Selected miRNAs Were Chosen Based on RNA-Seq Results: Two Were Common to Both Radiation Doses (*miR-99a-5p*, *miR-126a-3p*), Two Were Unique to the 100 mGy Group (*miR-133a-3p*, *miR-126a-5p*), and One was Specific to the 2 Gy Group (*miR-2137*). Expression was Normalized to Two Endogenous Control miRNAs, *miR-423-5p* And *miR-16-5p*. Bars Represent Mean ± SEM Relative to sham. Asterisks (*) Denote Statistically Significant Differences Relative to Sham (*P* < 0.05; One-Way ANOVA With Tukey’s Post Hoc Test), and Number Signs (#) Denote Significant Pairwise Differences Between the 100 mGy and 2 Gy Groups (*P* < 0.05; Tukey). RT-qPCR Results Corroborated the RNA-Seq Findings, Confirming the Direction and Magnitude of Differential miRNA Expression

To validate the NGS-based miRNAome findings, five representative miRNAs were selected for RT-qPCR analysis based on their miRNAome sequencing expression profiles. These included two miRNAs common to both radiation dose groups (miR-99a-5p, miR-126a-3p), two miRNAs specific to low-dose (miR-126a-5p, miR-133a-3p), and one unique to high-dose (miR-2137). Overall, the expression trends for all five miRNAs matched the direction and relative magnitude observed in the miRNAome sequencing data (Figure 4). Notably, miR-99a-5p and miR-126a-3p were significantly elevated in both 100 mGy and 2 Gy groups relative to sham, mirroring their robust and consistent induction observed in the NGS analysis. Among the low-dose–associated candidates, miR-126a-5p and miR-133a-3p were significantly upregulated at 100 mGy vs sham; moreover, both miRNAs showed significant between-dose differences (100 mGy >2 Gy; #, *P* < 0.05), aligning with the results presented in Figure 3B. Finally, miR-2137, which was selectively induced in the 2 Gy group by miRNAome analysis, also showed significant upregulation relative to sham in the RT-qPCR analysis. Taken together, these RT-qPCR results corroborate the sequencing results and indicate that markers significant vs sham and also differing between dose groups (eg, miR-126a-5p and miR-133a-3p) are especially promising candidates for dose binning.

## Discussion

**Figure 5.**
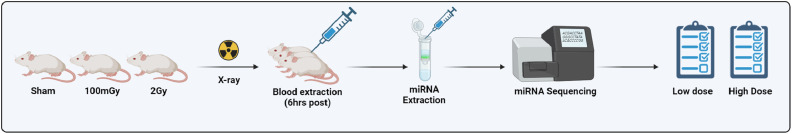
Schematic Overview of the in Vivo miRNAome Profiling Workflow for Radiation Biodosimetry. This Study Established a Streamlined and Scalable Pipeline for Unbiased Next-Generation Sequencing (NGS)-Based Profiling of the Circulating miRNAome Following Radiation Exposure. BALB/C Mice Were Exposed to Sham, 100 mGy, or 2 Gy X-Rays, and Blood was Collected 6 h Post-irradiation. Plasma was Isolated, and miRNAs Were Extracted Within 4 h Using a Silica Column-Based Method. Library Preparation and Indexing Were Completed in Approximately 8-10 h, Followed by Sequencing Using an Illumina Short-Read Platform Over a ∼24-h Run (2 × 100 bp Single-End). Bioinformatic Processing and Differential Expression Analysis Required an Additional 4-6 h. This Workflow Enables Rapid, high-Throughput Generation of Dose-specific miRNA Signatures, Supporting the Use of miRNA Profiling as a Minimally Invasive Tool for Radiation Biodosimetry. Created With https://BioRender.com

This study established a high-throughput, reproducible workflow for in vivo miRNAome profiling in a radiation exposure model, demonstrating its utility as a discovery platform for circulating miRNA biomarkers. As illustrated in Figure 5, the protocol leverages a streamlined sample-to-analysis pipeline that includes plasma isolation, RNA extraction, small RNA library preparation, NGS-based miRNAome profiling, and bioinformatic analysis, all within a 2-3-day window. By applying this pipeline to BALB/c mice exposed to 100 mGy or 2 Gy X-rays, we identified a total of 19 differentially expressed miRNAs for the high-dose condition and 7 for the low-dose condition. The inclusion of FDR <0.1 and a threshold of ≥50 transcripts per million (TPM) ensured analytical rigor in our differential expression analysis. Rather than serving only as a summary of experimental procedures, the workflow presented in Figure 5 is intended as a conceptual framework that can be broadly adopted by other groups for circulating miRNA biomarker discovery in radiation biodosimetry.

Our ability to detect and quantify low-abundant circulating miRNAs from 100 µL of plasma underscores the sensitivity of the approach. Further, RT-qPCR validation of five candidate miRNAs confirmed expression trends observed in the miRNA-seq dataset, supporting the robustness and reproducibility of the profiling workflow. These results validate our NGS-based method as a powerful tool for unbiased identification of radiation-induced miRNA signatures in small-volume biospecimens.

Previous literature examining blood-based miRNA responses to radiation exposure is notably sparse and largely focused on clinical or disease state models, such as radiation therapy patients or acute radiation injuries.^33-35^ Moreover, existing studies have frequently relied on candidate miRNA approaches or targeted PCR methodologies, potentially overlooking novel miRNAs that are critical to understanding systemic responses to radiation exposure. In contrast, our study employed an unbiased NGS-based miRNAome approach in a controlled, healthy murine model, allowing for comprehensive identification of circulating miRNAs uniquely responsive to radiation exposure. This underscores the need for further controlled and unbiased studies to robustly define baseline radiation-responsive miRNA signatures across various doses, exposure conditions, and timepoints.

Although relatively few studies have used unbiased NGS-based miRNA profiling in healthy murine models following ionizing radiation, several recent investigations using targeted or high-throughput approaches have reported overlapping plasma miRNA responses. For instance, both miR-150-5p and miR-155-5p, which were upregulated in our high-dose cohort, have been reported as significantly increased in mouse blood following total-body irradiation in multiple studies.^24,25,36^ Additionally, miR-99a-5p—identified in our study across both low and high doses—has been noted in a separate mouse survival model, where higher miR-100 family member levels, including miR-99a, coincided with reduced post-radiation survival.^37^ Although dose-time kinetics and experimental conditions vary widely, these convergent findings bolster confidence in the biological relevance of our miRNA hits and underscore the consistency of certain radiation-responsive miRNAs in vivo.

### Implications for Radiation Biodosimetry

Accurate and minimally invasive assessment of radiation exposure is essential for triage and response during radiological emergencies or accidental exposures. MiRNAs offer several advantages as biomarkers for biodosimetry, including high stability in plasma, dose- and time-dependent expression changes, and conservation across species.^7,8,26^ Our findings contribute to a growing body of evidence supporting miRNAs as radiation-responsive biomarkers. Specifically, the detection of both high-dose-specific and low-dose-specific miRNAs provides a dose-stratified profile that may aid in distinguishing between subclinical exposures and potentially harmful doses.

Although exposures around 100 mGy are not expected to cause overt clinical effects,^38-48^ their inclusion in this study is important because existing gold-standard biodosimetry methods lack sensitivity in this low-dose range.^1,6^ Demonstrating that plasma miRNA signatures can reliably capture biological responses to 100 mGy fills a critical gap by extending biodosimetry tools into subclinical exposure levels. In emergency scenarios, this capability would help distinguish between unexposed individuals, those with safe but biologically responsive low-dose exposures, and individuals requiring closer monitoring due to higher-dose exposures. Importantly, our data support the notion that low-dose exposures around 100 mGy may represent a biologically responsive yet clinically safe threshold where medical intervention may not be warranted, whereas higher doses such as 2 Gy are more likely to trigger systemic stress responses requiring clinical evaluation.

The observed overlap in miRNA expression between the 100 mGy and 2 Gy groups suggests that certain miRNAs (eg, miR-99a-5p, miR-126a-3p) may serve as general indicators of radiation exposure, while others (eg, miR-133a-3p or miR-2137) may reflect dose-specific biological responses. This layered signature approach could enhance biodosimetry toolkits by enabling classification of exposure severity.

Beyond dose-stratified signatures, translational conservation is essential for advancing these findings toward human biodosimetry. Most of the differentially expressed murine miRNAs identified in this study have well-established human orthologs, supporting their potential utility in clinical contexts.^49-51^ The mouse (mmu) differentially expressed miRNAs identified in this study and their corresponding human counterparts (hsa) include: mmu-miR-126a-5p (hsa-miR-126-5p), mmu-miR-126a-3p (hsa-miR-126-3p), mmu-miR-133a-3p (hsa-miR-133a-3p), mmu-miR-99a-5p (hsa-miR-99a-5p), mmu-miR-125a-5p (hsa-miR-125a-5p), mmu-miR-125b-5p (hsa-miR-125b-5p), mmu-miR-1a-3p (hsa-miR-1-3p), mmu-let-7e-5p (hsa-let-7e-5p), mmu-miR-151-3p (hsa-miR-151a-3p), mmu-miR-155-5p (hsa-miR-155-5p), mmu-miR-181a-5p (hsa-miR-181a-5p), mmu-miR-93-3p (hsa-miR-93-3p), mmu-miR-25-3p (hsa-miR-25-3p), mmu-miR-150-5p (hsa-miR-150-5p), and mmu-miR-451a (hsa-miR-451a). These are conserved across species and detectable in human plasma, reinforcing their translational potential. In contrast, mmu-miR-709^52^ and mmu-miR-2137^53^ appear rodent-specific, with no verified human orthologs, and may therefore have limited value as human biomarkers. These distinctions are important for prioritizing candidates: conserved miRNAs can be advanced toward human plasma testing, whereas rodent-specific species may still offer mechanistic insight into murine radiation responses but are unlikely to serve as universal biomarkers. Collectively, this conservation analysis underscores the strong translational potential of the majority of signatures identified in this study and supports their advancement toward human biodosimetry validation.

To enable practical implementation, future work should aim to incorporate these miRNA signatures into portable diagnostic formats such as isothermal amplification lateral flow assays or microfluidic biosensors for field deployment. Notably, recent advancements have demonstrated the feasibility of developing point-of-care lateral flow assays specifically tailored for miRNA detection.^54^

### Biological Relevance of Differentially Expressed miRNAs

The circulating miRNA profiles observed following exposure to low-dose (100 mGy) and high-dose (2 Gy) X-ray radiation revealed distinct and overlapping responses, suggesting different biological mechanisms and cellular origins of release into plasma. The miRNAs selectively upregulated at 100 mGy, miR-126a-5p and miR-133a-3p, likely reflect cellular signaling events distinct to low-level radiation exposures. MiR-126a-5p, with a notable fold-change of approximately 3.5, is predominantly endothelial-derived and is recognized as a biomarker for cardiovascular diseases, vascular injuries, and inflammatory responses.^55-57^ Its increased plasma levels could suggest endothelial activation or mild vascular stress responses to low-dose radiation. MiR-133a-3p, another low-dose–specific miRNA (fold-change ∼3.0), is typically expressed in cardiac and skeletal muscle tissue and has roles in tissue remodeling and muscle-specific injuries, along with serving as a biomarker in various cancers.^58,59^ Its induction at 100 mGy may indicate radiation-induced subtle stress responses in muscular or cardiac tissues, resulting in systemic release into circulation.

Five miRNAs were significantly elevated following both low- and high-dose exposures: miR-99a-5p, miR-125a-5p, miR-125b-5p, miR-1a-3p, and miR-126a-3p. MiR-99a-5p (fold-change ∼3.2-3.6), miR-125a-5p (∼2.5-2.6), and miR-125b-5p (∼2.6-2.8) belong to conserved miRNA clusters associated with hematopoietic lineage determination, tumor suppression, and DNA repair processes, implicating potential bone marrow or lymphoid tissue involvement.^17,58,60,61^ Their consistent elevation across both doses suggests activation of general hematopoietic stress responses or early DNA damage signaling. It is also important to note that because plasma was analyzed, circulating miRNA pools are naturally enriched for hematopoietic and immune-derived species. The prominence of lineage-associated miRNAs such as miR-99a-5p, miR-125a-5p, and miR-125b-5p may therefore reflect their cellular origin as much as their radiation responsiveness. This does not diminish their potential utility as circulating biomarkers, but highlights the need to interpret plasma-based signatures in light of tissue-of-origin effects.

Importantly, systemic signals were not limited to hematopoietic sources: miR-1a-3p (∼3.7-3.8), commonly recognized as a marker of cardiac tissue injury and mitochondrial dysfunction,^55,62^ suggests oxidative stress and muscular involvement, while miR-126a-3p (∼2.1-2.5), an endothelial-specific miRNA, aligns with vascular injury and inflammatory responses.^63,64^ Together, these findings indicate that plasma miRNA signatures represent a composite readout of radiation-induced stress across hematopoietic, cardiac, and vascular compartments.

The miRNAs uniquely upregulated at the high radiation dose (2 Gy) likely reflect acute cellular damage and inflammatory processes typically activated at higher radiation levels. Among these, miR-155-5p (∼3.6), a well-studied immune and inflammatory miRNA, has been associated with radiation-induced pulmonary fibrosis and inflammation,^65-67^ indicating possible immune-cell derived systemic inflammatory responses. Similarly, miR-2137 (∼3.2), previously identified as a biomarker for ischemic stroke,^53^ may reflect acute cerebrovascular or inflammatory stress responses triggered by HDR exposure. MiR-143-3p (∼2.9), involved in regulating ERK and AKT signaling through KRAS inhibition,^68-70^ could indicate stress-induced signaling from epithelial tissues or activated macrophages. MiR-709 (∼59.9), demonstrating the highest fold-change, is notably associated with regulating inflammatory responses and acute radiation syndrome, suggesting substantial systemic inflammatory activation or DNA methylation changes at higher radiation exposures.^23,52^ Additional miRNAs selectively induced at 2 Gy, such as miR-181a-5p (∼2.4), miR-151-3p (∼2.2), miR-let-7e-5p (∼3.0), and miR-150-5p (∼2.0), further highlight acute tissue-specific damage, including neural, muscular, immune, and inflammatory responses commonly observed following HDR exposure.^23,71-73^

Several miRNAs were significantly downregulated at 2 Gy, including miR-186-5p (−2.7 fold-change), miR-93-3p (−2.9), and miR-25-3p (−1.7), implicating potential suppression or selective degradation of these miRNAs from tissues including myocardial and tumor-derived tissues following acute stress.^74-77^ miR-451a (−1.9), commonly associated with tumor suppression and inflammation modulation, could reflect a systemic reduction related to acute cellular turnover or apoptosis following HDR exposure.^78,79^

Interestingly, our study identified an early increase in plasma miR-150-5p (∼2-fold at 6 h post-2 Gy), whereas several prior studies have reported decreases in circulating miR-150-5p following radiation, particularly at later timepoints (24-48 h) and after partial-body or higher-dose exposures.^23,80,81^ This discrepancy may reflect kinetic differences, with an early transient rise giving way to depletion during later hematopoietic injury phases. Differences in irradiation model (whole-body vs thoracic), dose, and strain background may also contribute. Taken together, these findings suggest that miR-150-5p expression may be dynamic and context-dependent, highlighting the importance of dose, model, and temporal resolution when interpreting miRNA biodosimetry candidates.

In addition to expression changes, the genomic organization of radiation-responsive miRNAs may provide insights into their regulation.^51^ Several of the identified candidates originate as 5p/3p strand pairs from the same precursor transcript, including miR-125a-5p/3p and miR-126a-5p/3p, which arise from *MIR125a* (chromosome 17 in mice) and *MIR126a* (chromosome 2 in mice), respectively.^50^ These paired products are co-regulated during processing of the same hairpin precursor, suggesting that radiation may influence their biogenesis in a coordinated manner.^9^ By contrast, all other differentially expressed miRNAs identified in this study are encoded at distinct loci distributed across the genome, consistent with independent regulation. This genomic distribution indicates that radiation-induced chromatin remodeling is unlikely to act on a single locus, but rather involves multiple dispersed regions, some producing coordinated 5p/3p strand responses and others functioning independently. Such heterogeneity supports a model where both local precursor regulation and broad transcriptional changes contribute to the systemic plasma miRNA signature.^82^

Overall, the miRNA signatures identified in this study suggest complex and dose-specific systemic responses to radiation exposure, reflecting tissue-specific injury, inflammation, hematopoietic activation, and oxidative stress responses. Low-dose responses appear to primarily reflect adaptive or mild stress signals from vascular and muscular systems, whereas high-dose miRNA profiles suggest acute inflammatory, immune, and cellular damage pathways. These findings underscore the potential utility of plasma miRNA profiling not only for radiation biodosimetry but also as a tool to better understand the underlying biological processes triggered by different radiation exposure scenarios.

### Study Limitations and Future Directions

Although our pipeline offers a sensitive and scalable approach for miRNA biomarker discovery, several limitations should be acknowledged. First, the study focused exclusively on a single timepoint (6 h post-irradiation), which limits temporal resolution of miRNA expression kinetics. This interval was chosen based on our prior in vitro work,^26^ where miRNA responses were evaluated at 1, 6, and 24 h post-radiation and the most robust expression changes were consistently observed at 6 h, while responses diminished substantially by 24 h. We therefore anticipated that 6 h would capture peak systemic changes in vivo. Nevertheless, it is likely that some miRNAs exhibit peak expression at earlier or later intervals that were not captured in this design. Second, the analysis was limited to two radiation doses—an acute low-dose (100 mGy) and a high-dose (2 Gy)—using a single radiation source. Additional studies are needed to assess the impact of dose rate, radiation quality (eg, gamma, proton, neutron), and chronic vs acute exposure paradigms on miRNA signatures.

Radiation sensitivity is also known to vary substantially between mouse strains, which has important implications for dose selection and biological interpretation.^38^ BALB/c mice, used in this study, are considered relatively radiosensitive, with an LD_50/30_ of approximately 5000 mGy, compared to more radioresistant strains like C57Bl/6, which have an LD_50/30_ closer to 6300 mGy.^83^ These strain-specific differences must be taken into account when extrapolating preclinical findings to human contexts, where the estimated LD_50/30_ is approximately 4500 mGy.^30^ Future validation of candidate miRNA signatures in larger cohorts, across multiple strains, and in human biospecimens will be essential to ensure robustness and translational relevance. Thus, additional functional studies are warranted to determine whether the differentially expressed miRNAs serve solely as exposure indicators or actively contribute to modulating radiation response pathways.

In addition, while only a subset of five representative miRNAs were validated by RT-qPCR in this study, all differentially expressed candidates identified by sequencing could, in principle, be subjected to independent verification. Expanding RT-qPCR validation to include the full set of dose- and time-responsive miRNAs will be an important next step, particularly as future studies incorporate broader dose ranges and multiple timepoints.

Finally, both discovery (NGS) and technical validation (RT-qPCR) were performed in the same animals (n = 5 per group). This within-cohort approach minimizes inter-animal variability and facilitates cross-platform concordance but does not provide independent biological replication. Accordingly, dose-specific findings, especially the low-dose–responsive candidates will be further validated in future studies with an independent cohort with increased sample size and/or orthogonal assays.

Despite these limitations, this study establishes a foundational framework for miRNA-based radiation biodosimetry and demonstrates the feasibility of integrating transcriptomic profiling with practical diagnostic applications.

## Conclusion

This study demonstrates a streamlined, unbiased NGS approach for in vivo profiling of circulating miRNAs in response to ionizing radiation. By identifying distinct and overlapping miRNA signatures at low (100 mGy) and high (2 Gy) doses, we establish a robust framework for miRNA-based biodosimetry. These findings support the potential of circulating miRNAs as minimally invasive biomarkers for radiation exposure, with implications for rapid dose assessment and future point-of-care diagnostic development.
